# *Melissa officinalis* L. (Lemon Balm): An Integrative Review of Phytochemistry and Evidence from Preclinical Research to Clinical Studies

**DOI:** 10.3390/plants15040650

**Published:** 2026-02-19

**Authors:** Ioan-Alexandru Cîmpeanu, Casiana Boru, Cristina Adriana Dehelean, Sergio Liga, Raluca Mioara Cosoroabă, Simona Ardelean, Roxana Popescu, Daliborca Vlad

**Affiliations:** 1 Doctoral School, “Victor Babes” University of Medicine and Pharmacy, Eftimie Murgu Square 2, 300041 Timișoara, Romania; ioan.cimpeanu@umft.ro; 2 Faculty of Medicine, “Vasile Goldiș” Western University of Arad, 86 Liviu Rebreanu Street, 310048 Arad, Romania; boru.casiana@uvvg.ro; 3 University Clinic of Toxicology, Drug Industry, Management and Legislation, Faculty of Pharmacy, “Victor Babes” University of Medicine and Pharmacy, 2nd Eftimie Murgu Square, 300041 Timișoara, Romania; cadehelean@umft.ro; 4 Research Centre for Pharmaco-Toxicological Evaluation, Faculty of Pharmacy, “Victor Babes” University of Medicine and Pharmacy, 2nd Eftimie Murgu Square, 300041 Timișoara, Romania; 5 Department of Applied Chemistry and Engineering of Organic and Natural Compounds, Faculty of Chemical Engineering, Biotechnologies and Environmental Protection, Politehnica University Timisoara, Vasile Pârvan No. 6, 300223 Timișoara, Romania; 6 Faculty of Dental Medicine, “Victor Babes” University of Medicine and Pharmacy, Revolutiei Ave. 1989, No. 9, 300580 Timișoara, Romania; cosoroaba.raluca@umft.ro; 7 Department of Pharmaceutical Technology, Faculty of Pharmacy, “Vasile Goldiș” Western University, 86 Liviu Rebreanu, 310045 Arad, Romania; ardelean.simona@uvvg.ro; 8 Department of Cell and Molecular Biology, Faculty of Medicine, “Victor Babes” University of Medicine and Pharmacy, 2nd Eftimie Murgu Square, 300041 Timișoara, Romania; popescu.roxana@umft.ro; 9 Biochemistry and Pharmacology Department, Discipline of Pharmacology, “Victor Babes” University of Medicine and Pharmacy, 300041 Timișoara, Romania; vlad.daliborca@umft.ro

**Keywords:** *Melissa officinalis*, lemon balm, Lamiaceae family, rosmarinic acid, clinical trials, bibliometric analysis

## Abstract

*Melissa officinalis* L. (lemon balm) is a Lamiaceae species widely used in traditional and contemporary herbal practice, yet its reported bioactivities are strongly preparation-dependent, reflecting variability between polyphenol-rich extracts and volatile essential-oil fractions. This integrative review links phytochemistry with recent preclinical findings and available clinical evidence. Across model systems, lemon balm most consistently shows antioxidant and anti-inflammatory signatures, with additional domain-specific signals reported in neurobehavioral, cardiometabolic, gastrointestinal, and dermatological models; however, comparability is limited by heterogeneous plant parts, extraction procedures, and chemical standardization. Preclinical findings were organized by biological domain, whereas clinically, the most consistent signals are observed for symptom-oriented endpoints, particularly anxiety/stress and sleep-related outcomes reported in controlled trials, including aromatherapy studies, while evidence for other indications remains mixed or insufficiently confirmed. Overall, the evidence supports continued development of chemically characterized, standardized preparations and mechanism-informed trials with harmonized outcomes and robust safety reporting to improve translational interpretability.

## 1. Introduction

Traditional healing systems and contemporary natural products research still depend on medicinal and aromatic plants, which continue to provide structurally diverse bioactive compounds with broad therapeutic potential [1,2]. The sustainability of plant-based therapeutics, particularly in regions with long-established medical traditions, is reflected by the significant reliance on herbal medicine on a global scale [2]. Historical use of medicinal plants was a result of cumulative trial and error experience and the transmission of traditional knowledge between generations, which later inspired systematic efforts to turn empirical claims into evidence-based arguments [1]. The biological activity of medicinal and aromatic plants is typically attributed to specialized molecules (e.g., polyphenols, essential oils), but their qualitative and quantitative profiles can significantly differ based on the plant component, geographical location, and processing or extraction conditions [3,4]. To support safe and effective clinical integration of medicinal plants, robust quality control and standardization are necessary due to inherent variability, toxic constituents, or non-standardized traditional practices [1,2,4,5].

**Figure 1:**
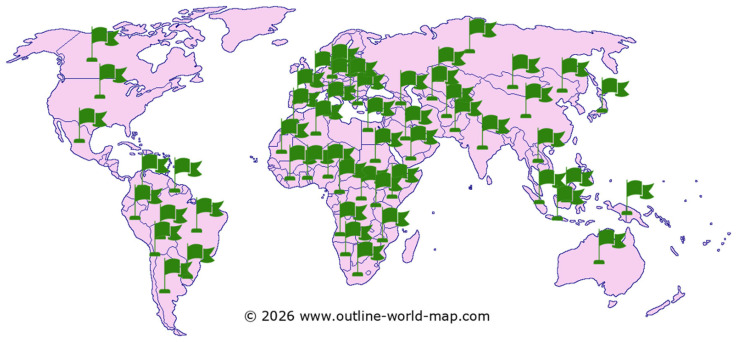
World map distributions of the Lamiaceae family. The image was obtained and modified from the Outline World Map (free access https://www.outline-world-map.com, accessed on 14 January 2026).

The Lamiaceae (Labiateae) family is a significant group of medicinal and aromatic plants, with diverse applications in both traditional medicine and the food, cosmetic, and pharmaceutical industries, which are distributed globally, with a particular focus on Mediterranean and subtropical regions (Figure 1) [3,4,6,7].

The Lamiaceae family’s pharmaceutical and industrial importance is largely attributed to its ability to produce high-value secondary metabolites, particularly essential oils and phenolic compounds, which have been repeatedly linked to numerous therapeutic activities in scientific research [7,8,9,10]. Table 1 provides a brief contextual overview of commonly reported Lamiaceae bioactivities and major phytochemical classes to situate *M. officinalis* within the family; detailed evidence for lemon balm is discussed in the following sections. Also, standardization and phytochemical reporting is the preferred method for interpreting the evidence base across Lamiaceae, as its composition is influenced by genetics, geography, cultivation, and post-harvest handling [7,8].

As summarized in Table 1, the medicinal and aromatic plants of the Lamiaceae family are characterized by a rich and diverse phytochemical profile, with essential oils and phenolic constituents recurring across multiple genera. Building on the broad relevance of Lamiaceae-derived phytochemical profiles and the well-recognized problem of compositional variability, a focused synthesis is needed for *Melissa officinalis* to connect preparation-dependent chemistry with domain-specific biological outcomes. Accordingly, the present review maps *M. officinalis* from botanical and phytochemical fundamentals through recent preclinical advances and available clinical evidence, with emphasis on endpoints that best support translation and reproducibility across study types.

## 2. Scope of the Review and Methodology

The scope of this review is to situate *Melissa officinalis* L. within the Lamiaceae family and to synthesize evidence linking its phytochemical profile to preclinical and clinical outcomes. Specifically, the review covers: (i) the phytochemical characteristics of *M. officinalis*; (ii) preclinical studies organized by biological domain; and (iii) clinical evidence and translational considerations.

This review adopts a five-stage bibliometric approach: (i) identification of the most relevant peer-reviewed literature; (ii) structured organization and cleaning of bibliometric records to ensure consistency and reliability; (iii) field-level analysis and processing of the dataset; (iv) synthesis and visualization of results to support interpretation; and (v) integration of key findings to generate conclusions and inform future research directions. In step (i), ‘most relevant’ records were defined as peer-reviewed publications retrieved by the stated search strategy that had *Melissa officinalis* as the primary focus (e.g., phytochemistry, preclinical/clinical outcomes), fell within the predefined time window, and provided sufficient methodological detail to extract the intervention type and outcomes for narrative synthesis. In step (ii), bibliometric ‘cleaning’ consisted of removing duplicate records across databases and harmonizing metadata fields (e.g., author names, journal titles, year).

The literature search was conducted in PubMed (https://pubmed.ncbi.nlm.nih.gov, accessed on 9 January 2026), Scopus/ScienceDirect (https://www.sciencedirect.com, accessed on 9 January 2026), MDPI (https://www.mdpi.com, accessed on 9 January 2026), and Google Scholar (https://scholar.google.com, accessed on 9 January 2026), covering publications from January 2020 to December 2025. These sources were selected for their broad multidisciplinary coverage, which supports the robustness and reproducibility of bibliometric analyses. Full metadata were exported for all eligible records, including titles, abstracts, author keywords, and reference lists. The search strategy used the following keywords and combinations: “Lamiaceae family”, “Lamiaceae biological activities”, “*Melissa officinalis*”, “lemon balm”, and “lemon balm phytochemistry”. To capture complementary and foundational evidence, reference lists were also screened to identify landmark studies published before 2020 that were scientifically pivotal for the field.

**Figure 2:**
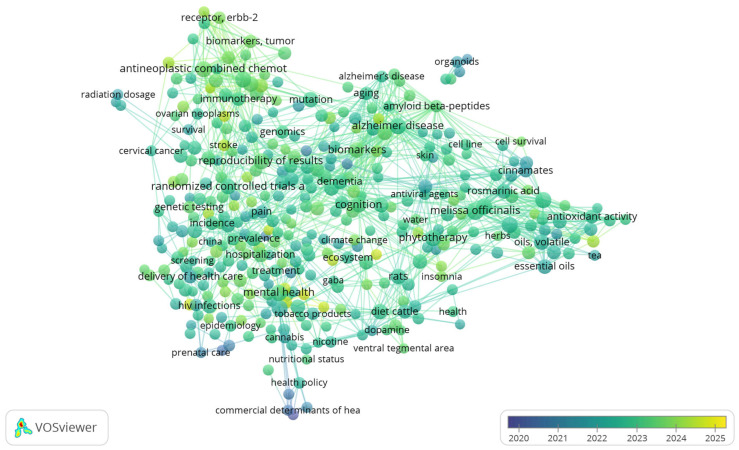
Co-keyword occurrence network (overlay visualization) constructed in VOSviewer from all keywords of the retrieved scientific publications (2020–2025).

A keyword co-occurrence analysis was conducted to identify thematic structures and research trends in scientific publications related to *Melissa officinalis* L. Terms were extracted from the titles, abstracts, and author keywords of all selected documents. As part of the synthesis and visualization stage, a co-keyword occurrence map was generated in VOSviewer v1.6.20 (www.vosviewer.com, accessed on 9 January 2026), from a PubMed dataset comprising 1670 publications. In this network visualization, node size reflects keyword frequency, links indicate co-occurrence within the same record and overlay colors represent the average publication year (2020–2025). The resulting map was used to support scoping decisions and inform the structure of the subsequent narrative synthesis. In the co-occurrence network, *Melissa officinalis* is most closely associated with keywords related to phytochemistry- and bioactivity-related terms (e.g., polyphenols/phenolics, antioxidant activity, antiviral agents, essential oils or volatile oils), as well as with biomedical themes aligned with the review’s scope. In addition, the overlay visualization shows that there has been relatively little research activity on lemon balm during the 2020–2025 period. Because the analysis is based on all keywords, the network also includes broader indexing terms; therefore, Figure 2 is intended for thematic scoping and trend mapping rather than causal inference.

## 3. *Melissa officinalis* L. (Lemon Balm): Botanical Aspects and Phytochemical Profile

The biological activity attributed to *Melissa officinalis* is closely tied to its botanical identity and to pronounced variation in chemical composition driven by plant part, cultivation/harvest conditions, and especially preparation method. The following section therefore summarizes the key botanical traits of lemon balm and outlines the principal metabolite classes most consistently reported across extracts and essential oils, providing a foundation for comparing evidence across experimental systems and study designs.

### 3.1. Botanical Aspects

*Melissa officinalis* L. is an herb known for its perennial growth, bushy appearance, and aromatic scent, and it has been widely grown and naturalized beyond its Mediterranean origin [87,88,89]. In terms of morphology, the plant usually grows between 30 and 150 cm and has soft hairs on most of its aerial parts [89,90]. The stem is erect, branched, usually glabrous, and quadrangular, consistent with many Lamiaceae species [88]. The leaves come in pairs and are petiolate and ovate, usually two to eight cm long, with a cuneate upper surface and cordate base, and crenate/scalloped-toothed margins. In certain instances, leaves may have a subglabrous appearance and may exhibit glandular hairs or punctate glands on the underside [87,88,90]. The characteristic lemon-like aroma is attributed to the plant’s volatile constituents (e.g., essential oils) [89,91]. During the summer, flowers are typically white to pale pink and are arranged in small clusters of 4–12 blossoms. The species is characterized by cross-pollination with perfect (bisexual) flowers [87,88]. The plant’s reproductive system involves two stamens and a four-lobed ovary, which yields 1–4 nutlets. The seeds are very small (1–1.5 mm), ovate, and dark brown/black, and prolonged storage may decrease their germination vigor [85,86]. It thrives in soils that are both fertile and well-drained, such as sandy loam, and can grow in full sunlight or partial shade [87,88,92].

### 3.2. Emerging Phytochemicals Profile

**Figure 3:**
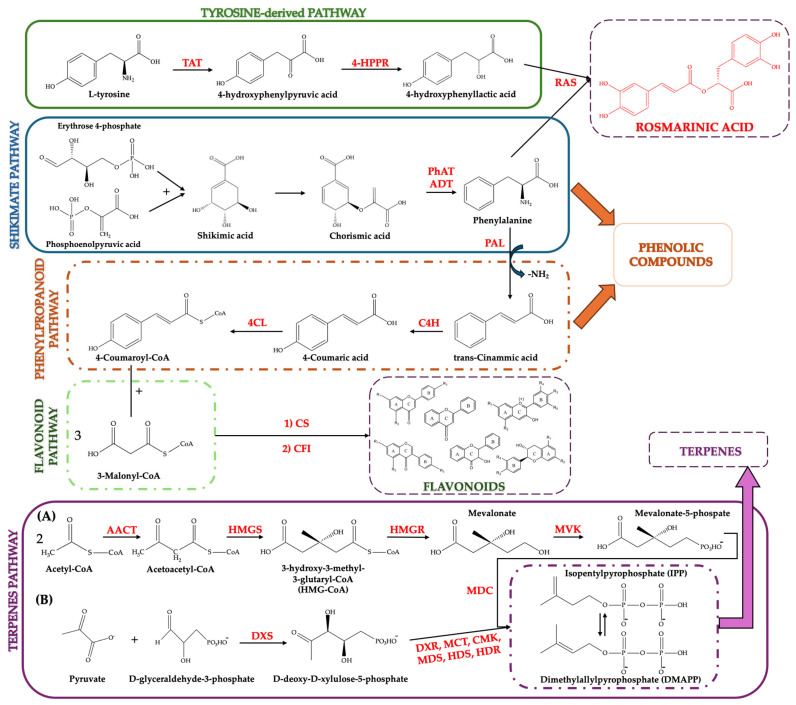
Schematic overview of major biosynthetic routes in *Melissa officinalis* L.

*Melissa officinalis* has been extensively studied in phytochemical studies, which demonstrate that it has a dual specialization in polyphenols (e.g., phenolic acids, flavonoids) and volatile terpenes, which together create its bioactive potential and distinctive aroma. In this subsection, the biosynthetic basis of emerging metabolites is outlined, and Figure 3 illustrates an integrative framework connecting precursor supply routes with the diversification of bioactive compounds.

Tyrosine-derived biosynthesis proceeds through tyrosine aminotransferase (TAT)-mediated formation of 4-hydroxyphenylpyruvate, hydroxyphenylpyruvate reductase (HPPR)-catalyzed reduction to 4-hydroxyphenyllactate, and subsequent rosmarinic acid synthase (RAS)-catalyzed ester formation with a hydroxycinnamoyl-CoA donor to produce rosmarinic acid [91,93]. The phenylpropanoid pathway is initiated from the amino acid phenylalanine, which is converted by phenylalanine ammonia-lyase (PAL) into trans-cinnamic acid through deamination. Trans-cinnamic acid is then hydroxylated by cinnamate 4-hydroxylase (C4H) to produce 4-coumaric acid. Subsequently, 4-coumarate-CoA-ligase (4CL) activates 4-coumaric acid to form 4-coumaroyl-CoA, a key metabolic node that feeds the synthesis of numerous phenolic compounds and serves as the gateway precursor for flavonoid biosynthesis [91,93,94]. Flavonoids constitute another major axis of the phytochemical profile, originating from phenylpropanoid metabolism and branching into structurally and functionally distinct subclasses whose accumulation is shaped by enzyme availability and multilayer regulation at the transcriptional level [94,95,96,97,98]. Flavonoid biosynthesis is initiated when chalcone synthase (CS) condenses p-coumaroyl-CoA with three malonyl-CoA units to form a chalcone, which chalcone–flavanone isomerase (CHI) then isomerizes to the flavanone that branches into multiple flavonoid classes [94,96,97]. Terpenoid biosynthesis in plants relies on two compartmentalized pathways that generate the universal isoprene C_5_ precursors isopentenyl diphosphate (IPP) and dimethylallyl diphosphate (DMAPP). In the cytosol, the mevalonate (MVA) pathway converts acetyl-CoA through sequential steps catalyzed by acetyl-CoA acetyltransferase (AACT), HMG-CoA synthase (HMGS), and HMG-CoA reductase (HMGR), followed by mevalonate kinase (MVK) and downstream reactions leading to IPP [94,95,96]. In plastids, the methylerythritol phosphate (MEP) pathway is initiated by D-deoxy-D-xylulose-5-phosphate synthase (DXS) and proceeds via 1-Deoxy-D-xylulose-5-phosphate reductoisomerase (DXR), 2-Methyl-D-erythritol-4-phosphate cytidylyltransferase (MCT), 4-(Cytidine-5′-diphospho)-2-methyl-D-erythritol kinase (CMK), 2-Methyl-D-erythritol 2,4-cyclodiphosphate synthase (MDS), 4-Hydroxy-3-methylbut-2-enyl diphosphate synthase (HDS), and 4-Hydroxy-3-methylbut-2-enyl diphosphate reductase (HDR) to yield IPP and DMAPP. These pathways provide a biochemical rationale for the co-occurrence of diverse mono-, sesqui-, and diterpenes in lemon balm essential oil profiles [91,98,99,100].

Lemon balm exhibits a chemically diverse phytochemical composition that underpins both its sensory attributes and its reported biological activities. To facilitate a structured interpretation of this diversity, the major constituents are grouped into four complementary categories (e.g., volatile compounds, polyphenolic compounds, terpenes, other phytochemicals) reflecting both their chemical nature and their biosynthetic origin. These constituents are primarily accumulated in glandular trichomes and are typically analyzed as part of the essential oil fraction. The volatile profile is dominated by oxygenated monoterpenes and their derivatives (e.g., citronellal, neral, geranial), although sesquiterpenes (e.g., (E)-caryophyllene, caryophyllene oxide) and minor non-terpenoid volatiles are also reported [90,101,102].

Across the non-volatile fraction, lemon balm is especially recognized for hydroxycinnamic acids, with rosmarinic acid (RA) widely used as a quality-control biomarker because of its typically high abundance relative to other constituents [102,103,104]. In a separate valorization study of *M. officinalis* distillation by-products, high-performance liquid chromatography coupled with diode-array detector (HPLC-DAD) analysis identified rosmarinic acid as the major phenolic component of the aqueous extract (reported at 73.5 mg/g dry extract), underscoring that RA remains highly recoverable even after essential oil distillation [105].

Beyond phenolic acids, lemon balm contains a broad spectrum of flavonoids (e.g., quercetin, kaempferol, apigenin) and additional secondary metabolites, including tannins, coumarins, and polysaccharides, which collectively contribute to antioxidant and bioactive properties attributed to the herb [103,104]. The triterpenoid fraction includes ursolic acid and oleanolic acid as key representatives in the plant’s phytochemical repertoire. In addition, a detailed phytochemical study on polar metabolites from the aerial parts led to the isolation and structural elucidation of new ursene triterpene glycosides (e.g., melissiosides A-C), along with a related sulfated triterpenoidal glycoside (e.g., 23-sulfate ester of nigaichigoside F1), highlighting that lemon balm contains not only free triterpenes but also chemically complex glycosylated/sulfated triterpenoids [106].

The dual specialization of lemon balm in polyphenols (notably hydroxycinnamic acids and flavonoids) and volatile terpenoids provides a plausible basis for its multi-target biological profile, but also introduces a key interpretive challenge, as studies frequently use non-equivalent preparations, doses, and standardization approaches. To maintain interpretability, the preclinical evidence is therefore organized by biological domain, with emphasis on outcomes that most directly inform mechanism and translational relevance.

## 4. Recent Advances in Preclinical Research

Preclinical studies on *Melissa officinalis* are characterized by substantial heterogeneity in botanical preparations (aqueous or hydroalcoholic extracts versus essential oils), phytochemical standardization, and experimental dosing paradigms, which may limit direct comparability across models. Furthermore, many findings derive from in vitro systems or short-duration animal studies in which exposure levels may not reflect achievable human pharmacokinetics. Accordingly, the key studies are summarized below by biological domain to support mechanistic interpretation, with conclusions contextualized in terms of translational relevance.

### 4.1. Central Nervous System and Neurobehavioral Domain

Recent reviews frame the neurobehavioral relevance of *Melissa officinalis* as a function of its multi-constituent phytochemical matrix, emphasizing that polyphenol-rich extracts, often standardized to rosmarinic acid, and the volatile essential oil (e.g., citral isomers geranial and neral) may engage partially distinct neurogical pathways [107].

Although diabetes is primarily a metabolic disorder, it is frequently associated with cognitive deficits and hippocampal molecular alterations via mechanisms such as oxidative stress, inflammation, and vascular dysfunction; therefore, we include studies when the primary outcomes are neurobehavioral and CNS-relevant. Within the cognitive domain, Naseri et al. evaluated a hydroalcoholic *M. officinalis* extract in a streptozotocin-induced diabetic rat model and incorporated both behavioral and molecular endpoints. Animals were allocated to control/diabetic groups and treated with *M. officinalis* (25, 50, or 100 mg/kg) for two weeks; learning and memory were assessed using Y-maze and passive avoidance paradigms, followed by hippocampal qPCR. The extract’s phytochemical profiling identified rutin as the main flavonoid and rosmarinic acid as the main phenolic compound. Behaviorally, *M. officinalis* improved Y-maze alternation (most pronounced at 50 mg/kg) and increased step-through latency in passive avoidance (all doses), indicating partial reversal of diabetes-associated cognitive impairment. At the molecular level, diabetes reduced hippocampal BDNF and nitric oxide synthase (NOS) gene expression, whereas extract treatment increased these transcripts relative to untreated diabetic rats (*p* < 0.05), aligning behavioral improvement with neuroplasticity- and NO-signaling-related pathways [108]. In a metabolically distinct neurobehavioral model, Hatami et al. examined whether *M. officinalis* could mitigate high-fat-diet-associated behavioral changes. They used a lemon balm hydroalcoholic (30% ethanol) macerate (12 h, 22 °C + 12 h at 35 °C), followed by filtration, low-temperature solvent removal (<35 °C), and freeze-drying. Male Wistar rats were fed normal diet or HFD for five weeks, then treated daily for two further weeks with *M. officinalis* extract (50, 100, or 150 mg/kg, intraperitoneal) or vehicle; outcomes included sucrose preference (anhedonia), open field (locomotion), elevated plus maze (anxiety), Y-maze (working memory), and Morris water maze (spatial memory), alongside metabolic parameters and serum BDNF. HFD feeding induced obesity, neurobehavioral alterations (anhedonia/anxiety/depression-like patterns), memory deficits, and reduced serum BDNF, whereas extract administration—particularly at 100–150 mg/kg—was reported to improve neurobehavioral and cognitive performance in parallel with increased serum BDNF and improved metabolic indices (e.g., reduced weight gain and fasting glucose). Collectively, these data support a model in which lemon balm may modulate neurobehavioral outcomes in metabolically stressed animals, with BDNF regulation proposed as a mechanistic correlate rather than a confirmed causal mediator [109].

Beyond behavioral endpoints, mechanistic neuroprotection has been explored under hypoxic stress. Bayat et al. investigated a commercial *M. officinalis* balm oil (Balm Oil, Sigma B4008, Sigma, Bucharest, Romania) in both an in vitro neuronal hypoxia paradigm and an in vivo transient hippocampal ischemia model (two-vessel occlusion for 20 min in male rats). In primary cortical neurons exposed to 24 h hypoxia (5% O_2_) plus reperfusion, a 10 μg/mL dose reduced cell death markers relative to untreated hypoxic cultures, while higher concentrations showed toxicity in normoxia and were therefore avoided. In vivo, ischemia increased hippocampal caspase-3-like activity, produced numerous TUNEL-positive neurons in CA1, and caused marked neuronal loss on cresyl violet staining; treatment with lemon balm (reported at 100 mg/kg) significantly attenuated caspase activity and reduced apoptotic labeling and neuronal damage. The authors also reported reduced lipid peroxidation (malondialdehyde) and attenuation of ischemia-associated decrements in antioxidant capacity, alongside modulation of ischemia-responsive transcripts (including suppression of HIF-1α expression). Together, the findings are consistent with antioxidant/anti-apoptotic effects under hypoxic–ischemic conditions, while remaining limited to short-term experimental endpoints and a single injury context [110].

Finally, the CNS relevance of *M. officinalis* essential oil depends strongly on chemotype and constituent profile. While such compositional data help rationalize potential CNS bioactivity hypotheses (e.g., via volatility, olfactory pathways, or receptor interactions), translation across studies requires careful matching of preparation type, dosing route, and chemical standardization.

### 4.2. Metabolic Field

In a preclinical study conducted by Lee et al., an activity-guided *Melissa officinalis* leaf fraction (abbreviated ALS-L1023) was administered as 0.4% or 0.8% (*w*/*w*) dietary supplementation to HFD-fed female C57BL/6J mice for 16 weeks. Within this model, ALS-L1023 reduced body-weight gain, total/visceral adipose tissue mass, and visceral adipocyte size without a significant change in food intake versus obese controls. The same study reported improvements in systemic metabolic control, including reductions in circulating lipids and improvements in indices of insulin sensitivity (e.g., QUICKI/HOMA-IR) and glycemic excursion during OGTT relative to untreated obese mice. Consistent with improved glucose–insulin homeostasis, ALS-L1023 was associated with lower fasting glycemia and insulinemia (reported for the higher-dose condition) and with attenuation of obesity-associated pancreatic changes, including reductions in islet hypertrophy and normalization of insulin-positive β-cell area toward lean controls [111].

In a separate diet-induced obesity model conducted by Kim et al., female C57BL/6 mice fed with an HFD received lemon balm extracts prepared in distilled water (LBD) or 80% ethanol (LBE) at 200 mg/kg/day for 6 weeks, with both extract groups showing a significantly lower weight-increase ratio than HFD controls. The extract exposure was linked to reduced mesenteric white adipose tissue (mWAT) and downregulation of adipose fatty-acid synthase (FAS) expression, with solvent-dependent differences in PPAR-γ/FAS patterns across depots. The authors also reported an apparent BAT-related component for the ethanol extract, noting increased BAT weight and higher PGC1-α protein levels despite weaker or discordant mRNA changes, which they interpreted as potentially consistent with altered thermogenic capacity [112]. In an HFD-rat paradigm, intraperitoneal hydroalcoholic extract administration (50–150 mg/kg/day for 2 weeks) was associated with reductions in weight gain, fasting blood glucose, and selected lipid parameters, with larger effects generally reported at higher doses. Separately, in diet-induced hypercholesterolemia, administration of *M. officinalis* as a fresh infusion in drinking water was associated with a reduction in total cholesterol compared to HFD alone, with additional lipid-fraction differences reported in the study [113].

### 4.3. Cardiovascular Field

In a study conducted by Draginic et al., thirty-two Wistar rats were allocated to a myocardial ischemia/reperfusion (I/R) control group or to pretreatment with an ethanolic *Melissa officinalis* extract (e.g., 50, 100, 200 mg/kg/day, orally) for 7 days prior to ex vivo global I/R injury. The extract was prepared from dried leaves using 70% ethanol under reflux (78.5 °C, 2.5 h), followed by filtration and rotary vacuum evaporation (40 °C, 90 rpm, 250 mbar) to obtain a dry extract. Following pretreatment, isolated hearts were perfused using the Langendorff technique and subjected to 20 min of global ischemia followed by 30 min of reperfusion, with cardiodynamic function recorded across the protocol. Oxidative stress indices were quantified both in coronary effluent (including superoxide anion, hydrogen peroxide, TBARS, and nitrite) and in heart tissue homogenate (e.g., TBARS, nitrite, SOD, catalase), and histology was used to evaluate myocardial architecture and collagen content (fibrosis). The authors reported that extract pretreatment improved cardiodynamics parameters and preserved myocardial architecture after I/R, with the most consistent effects observed at 200 mg/kg. In parallel, higher-dose pretreatment (100–200 mg/kg) reduced pro-oxidant readouts (e.g., TBARS and reactive oxygen species-related measures) and increased nitrite, consistent with modulation of oxidative stress and NO-related pathways in this model [114]. Also, they summarized preclinical and translational evidence suggesting that *Melissa officinalis* extracts and constituent phytochemicals may influence multiple cardioprotective pathways, with oxidative stress reduction and endothelial/NO signaling recurring across experimental models [115].

In a study conducted by Merimi et al., an in silico workflow was used to evaluate rosmarinic acid and representative flavonoids against hypertension-related protein targets, combining DFT optimization, molecular docking (MOE), and ADMET/drug-likeness predictions. Docking analyses reported comparative scores (kcal/mol) for ACE, AT1R, and PDE5, showing that rosmarinic acid and the flavonoid subset each yielded favorable docking scores, while the combined rosmarinic acid–flavonoids condition showed the strongest docking scores across targets (e.g., ACE −8.5, AT1R −8.8, PDE5 −9.1), alongside reference drugs (e.g., Captopril, Losartan, Sildenafil) [116].

### 4.4. Oncology Domain

In an in vitro breast cancer model (MCF-7, 48 h), Motafeghi et al. evaluated silver nanoparticles (AgNP), graphene, and an Ag-graphene nanocomposite synthesized using an ethanolic extract of *Melissa officinalis*. The central outcome was a concentration-dependent reduction in MCF-7 growth/viability, with the Ag-graphene nanocomposite producing the strongest effect; at the highest tested concentration, growth inhibition reached 84.60%. Mechanistically aligned readouts showed an oxidative-stress signature, with intracellular ROS and lipid peroxidation (LPO/MDA) increasing (reported up to 74% and 70%, respectively) and glutathione decreasing (reported down to 16% of control at the highest combined concentration), consistent with stress-mediated cytotoxicity [117]. Using colorectal cancer cells (HCT116), Kuo et al. combined quantitative proteomics with functional validation to map the anticancer effects of a hot-water *Melissa officinalis* extract. Tandem mass tag (TMT) proteomics quantified 3465 proteoforms, revealing 67 upregulated and 54 downregulated proteins after MO exposure, with bioinformatic enrichment highlighting a dominant suppression of mitochondrial respiration/oxidative phosphorylation (OXPHOS). A key result was the coordinated downregulation of 21 electron transport chain (ETC) proteins, heavily enriched for Complex I components, alongside decreases in Complex II and IV proteins; these shifts were validated by Western blot reductions in representative OXPHOS complex markers (e.g., Complex I/II/IV proteins) in a dose-dependent manner. Functionally, MO (notably 375 μg/mL, 48 h) increased intracellular ROS, decreased mitochondrial membrane potential (JC-1 ratio), and elevated apoptosis (Annexin V/PI), linking mitochondrial dysfunction to cell death. Importantly, the ROS scavenger N-acetyl-L-cysteine (NAC, 5 mM) reduced ROS and rescued multiple phenotypes supporting a ROS-triggered mechanism upstream of ETC impairment and apoptotic death [118].

Thapa et al. provide a synthesis of preclinical evidence (2010–2024 period, 85 studies) on the anticancer potential of *Melissa officinalis*, emphasizing mechanistic convergence across in vitro and in vivo models rather than a single experimental system. The review’s aggregated results indicate that MO and its phytochemicals (highlighting polyphenols such as rosmarinic acid and flavonoids such as luteolin) repeatedly associate with suppression of PI3K/Akt and MAPK/ERK signaling, induction of cell-cycle arrest, modulation of oxidative stress responses, and activation of apoptosis pathways (often framed through Bax/Bcl-2 balance and caspase engagement). Across the summarized studies, notable cytotoxic/anti-proliferative activity is reported in several frequently used cancer cell models (including PC-3, HT-29, and MCF-7), leading the authors to position MO as a candidate for adjuvant exploration while underscoring the need for further translational and clinical validation [119].

### 4.5. Gastrointestinal Field

In a study conducted by Juee et al., an ethanolic lemon balm leaf extract (250 or 500 mg/kg, p.o.) was evaluated in rats with absolute ethanol-induced gastric injury, using omeprazole (20 mg/kg) as a reference drug. They used lemon balm aerial parts and prepared an 80% ethanolic extract by ultrasound-assisted extraction (500 g dried at 1000 mL of 80% ethanol), followed by rotary evaporation at 40–50 °C to obtain a dry extract (yield: 18.21% *w*/*w*). The extract reduced ulcer severity in a dose-dependent manner, with the 500 mg/kg group showing a low ulcer index and a high prevention percentage relative to the negative control; gastric defensive parameters were also improved, including increased gastric mucus weight and higher gastric content pH versus controls. Histopathological assessment indicated reduced mucosal disruption/erosion, inflammatory cell infiltration, and submucosal edema in treated animals, particularly at the higher dose. Mechanistically, the extract was associated with lower gastric TNF-α and IL-1β compared with negative controls (and, for several comparisons, relative to the positive-control condition), consistent with attenuation of ethanol-triggered inflammatory signaling. The authors also reported in vitro antioxidant activity (DPPH EC50 in the low µg/mL range) and quantified tannin/flavonoid-related metrics, which were discussed as potentially relevant to mucosal protection [120].

In a study conducted by Dolatabadi et al., a hydroalcoholic *Melissa officinalis* extract was tested in rats across two IBS-relevant paradigms (acetic-acid-induced post-inflammatory hypersensitivity and restraint-stress-induced bowel dysfunction), with a mechanistic focus on nitrergic signaling. They prepared a hydroalcoholic extract by room-temperature maceration (200 g powdered material extracted with 600 mL of 70% ethanol for 72 h at 24 ± 3 °C), followed by filtration and evaporation under reduced pressure (reported yield: 23.5%), and stored the extract at −18 °C. In the acetic-acid model, oral administration (100–300 mg/kg/day for three days) improved visceral pain-related outcomes at the highest dose (300 mg/kg), including restoration of the nociceptive threshold (AWR score 2) toward sham values and reduced AWR scores at intermediate distension volumes. In the restraint-stress model, the same 300 mg/kg dose reduced stress-induced defecation frequency and shifted stool form toward harder pellets. At the tissue level, 300 mg/kg decreased colonic TNF-α and MPO activity, reduced lipid peroxidation (TBARS), and increased total antioxidant capacity (FRAP), indicating anti-inflammatory and antioxidant effects in stressed/irritated bowel tissue. Co-administration of NOS pathway inhibitors (L-NAME and/or aminoguanidine) attenuated the protective patterns (behavioral and biomarker outcomes), which the authors interpreted as supportive of NO-pathway involvement [121].

In a study conducted by Posłuszny et al., a commercial standardized *Melissa officinalis* leaf extract (Nor-Balm) was assessed for effects on intestinal contractility. Across 0.001–0.1 mg/mL, the extract increased acetylcholine-evoked contraction amplitude in both proximal and distal jejunum segments, indicating a net pro-contractile effect under the tested conditions. When examined as isolated constituents, phenolic acids (including rosmarinic and chlorogenic acid) predominantly reduced acetylcholine-induced responses, suggesting that the whole-extract effect could not be attributed to a single tested phenolic component and may reflect matrix interactions or non-phenolic constituents. The authors emphasized that divergent findings relative to the commonly cited “spasmolytic” profile of lemon balm may reflect differences in extract standardization, species physiology, and experimental setup [122]. Another study, conducted by Posłuszny et al., evaluated the same standardized extract, and selected phenolic acids were evaluated in the ovine jejunum and colon. In contrast to the broiler data, the extract and several phenolic acids (e.g., rosmarinic acid) generally reduced spontaneous contractility, while chlorogenic acid displayed more variable, tissue- and layer-dependent actions. Across preparations, acetylcholine-induced contractions were predominantly decreased, supporting an overall myorelaxant tendency in this ruminant model. Taken together, the two ex vivo datasets highlight that the direction of motility modulation may be species- and tissue-context dependent, reinforcing the need for careful standardization and model selection when extrapolating gastrointestinal pharmacology [123].

In a critical assessment conducted by Teterovska et al., lemon balm was identified as a frequent ingredient in food supplements marketed for gastrointestinal complaints; however, the authors emphasized that the substantiation of label health claims is often based largely on traditional use and preclinical rationale rather than consistent clinical validation, underscoring a translational gap between mechanistic plausibility and evidence thresholds required for health-claim support [124].

### 4.6. Dermatologic Domain

Sipos et al. investigated an aqueous *Melissa officinalis* leaf extract using an integrated phytochemical bioactivity workflow and a panel of skin-relevant outcomes spanning chemical profiling, cell-based assays, and an in vivo topical model. The extraction protocol was mixing 1 g dried, crushed lemon balm leaves with 100 mL distilled water for 48 h at 25 °C under orbital stirring (250 rpm), followed by sonication for 1 h (50% amplitude; 750 W), filtration (0.45 µm), and oven-drying at 40 °C to obtain a solid powder (re-dissolved for testing). Liquid chromatography coupled with mass spectrometry (LC–MS) quantification identified apigenin as the dominant quantified polyphenol (38.72 ng/mg dry weight), with substantially lower amounts of rutin (4.06 ng/mg d.w.), ferulic acid (1.25 ng/mg d.w.), chlorogenic acid (0.31 ng/mg d.w.), and caffeic acid (0.18 ng/mg d.w.), supporting an apigenin-rich phenolic signature for the extract. Functionally, the extract was well tolerated in HaCaT keratinocytes across 20–1000 µg/mL in an Alamar Blue readout, showing no cytotoxicity and, relative to a distilled-water control, a statistically significant increase in proliferation, consistent with a pro-viability/pro-regenerative profile in this non-malignant skin cell model. In contrast, in A375 melanoma cells, the extract displayed only a weak/selective cytotoxic signal in the authors’ interpretation, limited to the highest concentration tested (1000 µg/mL), suggesting a narrow therapeutic window for direct anti-melanoma activity under the specific conditions used. Extending these findings in vivo, topical application in SKH-1 hairless mice (5 mg/mL) was associated with improved skin biophysical parameters versus control/vehicle trajectories, including reduced transepidermal water loss (TEWL), decreased erythema, and increased hydration, collectively indicating a potential barrier-supportive and anti-irritant effect at the organism level [125]. In a study conducted by Pérez-Sánchez et al., a rosmarinic acid standardized lemon balm extract was investigated for UVB-related skin protection in HaCaT keratinocytes, with phytochemical profiling showing rosmarinic acid and salvianolic-acid derivatives as major compounds. During UVB treatment (800 or 1200 J/m^2^), LBE (15–100 mg/mL) increased keratinocyte survival, with significant protection starting at 80 mg/mL after 800 J/m^2^, and at 1200 J/m^2^ showing a significant effect only at 100 mg/mL, while rosmarinic acid alone showed non-significant trends in this setup. UVB increased intracellular reactive oxygen species (ROS) signal by approximately 33% (800 J/m^2^) and 45% (1200 J/m^2^) versus non-irradiated controls, while LBE reduced ROS versus irradiated controls by 19% (800 J/m^2^) and 23% (1200 J/m^2^) at 60 mg/mL, and by 29% (800 J/m^2^) and 33% (1200 J/m^2^) at 100 mg/mL. Using comet assay endpoints after 1200 J/m^2^, LBE at 100 mg/mL reduced UVB-induced DNA damage by 64% (and was described as non-genotoxic without UVB). UVB increased γ-H2AX activation by 16-fold, while LBE decreased γ-H2AX by roughly 50% at 15 mg/mL, showing a dose–response relationship that reached a plateau at higher concentrations [126]. In a study conducted by Jun et al., ethanolic extracts of *Melissa officinalis* (wild-type extract, MOE) and a UV-irradiated variant (UMOE) were examined for anti-melanogenic activity in B16-F1 murine melanocytes. The extraction protocol was washing, freeze-drying, and grinding the lemon balm leaves, followed by extraction in 80% (*v*/*v*) ethanol at room temperature for 24 h, filtration (Whatman No. 1), and freeze-drying of the filtrate to obtain dry extracts. In cells, UMOE reduced melanin content by 49% at 200 µg/mL. Expression of melanogenesis-related enzymes was downregulated, including tyrosinase, TRP-1, and TRP-2, with TRP-1 protein reported as reduced by 73% at 200 µg/mL UMOE [127].

Iwahashi et al. linked lemon balm extract activity to dermal extracellular-matrix remodeling by focusing on Endo180 (a collagen internalization/remodeling receptor) as a mechanistic target and then translating these findings toward topical use. The extraction protocol was the extraction of 200 g leaves with 3.2 L of 50% 1,3-butylene glycol at 80–90 °C for 3 days, followed by filtration, concentration under reduced pressure at 60 °C, and freeze-drying (reported yield 26%). In a comparative screen of 71 natural extracts using dermal fibroblasts, *Melissa officinalis* leaf extract (MOLE) at 50 µg/mL produced the strongest Endo180 induction, elevating Endo180 mRNA to 178.1% of control and increasing Endo180 protein to 127.4%, indicating that the extract robustly upregulated this remodeling-related axis at both transcriptional and protein levels under the assay conditions. The authors quantified rosmarinic acid as 10.2% of the extract and showed that rosmarinic acid alone (20 µg/mL) increased Endo180 production to 143.9% in whole-cell measurements, supporting the interpretation that this phenolic constituent contributes substantially—but may not fully account for—the extract’s activity. Functionally, in a UVB-associated paracrine damage paradigm (fibroblasts treated with conditioned medium from UVB-irradiated keratinocytes), MOLE at 200 µg/mL nearly completely prevented the UVB-conditioned-medium-driven decreases in Endo180 and type I collagen production, suggesting a protective effect on fibroblast matrix-remodeling capacity and collagen homeostasis under inflammatory/photodamage-relevant signaling [128].

Pressi et al. evaluated a cosmetic-oriented, in vitro plant-cell-culture-derived *Melissa officinalis* phytocomplex that was chemically standardized to ensure reproducibility, reporting rosmarinic acid at 7.6% (*w*/*w*) and total polyphenols at 9.2% (*w*/*w*, expressed as rosmarinic acid equivalents). The preparation protocol was filtration of the cell suspension (50 µm mesh), washing with 0.9% NaCl, addition of 1% (*w*/*w*) citric acid, homogenization (15,000 rpm, 20 min), and drying to obtain the phytocomplex powder. Functionally, the phytocomplex demonstrated an antioxidant, cell-protective signature in keratinocytes: in HaCaT cells challenged with H_2_O_2_, treatment at 0.1% reduced ROS generation relative to the oxidatively stimulated condition, indicating attenuation of stress-induced intracellular oxidative burden under the assay settings. Translating to a more tissue-relevant system, the authors used human skin explants pre-treated for six days and then exposed to high-dose blue light (445 ± 10 nm; 63.7 J/cm^2^), where blue-light exposure increased nuclear localization of Nrf2, while pre-treatment with 0.1% phytocomplex was reported to completely suppress this blue-light-induced increase in nuclear Nrf2 in a scoring-based histologic assessment, consistent with a reduction in blue-light-triggered oxidative stress signaling at the tissue level [129]. In a study conducted by Adamiak et al., fish type I collagen films were modified by incorporating a *Melissa officinalis* dry extract to generate a topical/cosmetic biomaterial matrix. The authors prepared collagen films by solvent evaporation and incorporated lemon balm extract at 29.62% (*w*/*w*) of the collagen–extract mixture. Importantly for dermatology-oriented applications, antioxidant capacity increased markedly in the collagen/Melissa films, with total antioxidant content rising from 0.62 ± 0.06 to 2.90 ± 0.23 mg Trolox equivalents/g, supporting the concept that lemon balm polyphenols can be retained in a collagen matrix while maintaining radical-scavenging potential. Overall, the study positions *M. officinalis* as a promising antioxidant functional additive for collagen-based cosmetic films, while also indicating a trade-off in mechanical performance that would be relevant for formulation design and intended skin-contact use [130].

Koushik et al. developed a *Melissa officinalis* essential oil-loaded nanoemulgel as a topical delivery system and optimized the formulation via a central composite design, obtaining a nanoemulsion with a mean droplet size of 127.31 nm, a low polydispersity (PDI 17.7%), and a zeta potential of −25.0 mV, collectively supporting colloidal stability and suitability for dermal application. The final nanoemulgel showed a skin-compatible pH (5.68 ± 0.07) and remained physically stable under accelerated storage (40 ± 2 °C; 75 ± 5% RH), exhibiting only minor viscosity shifts over 30 days, which is consistent with robustness for topical use and shelf-stability expectations. Biologically, antimicrobial activity was demonstrated by broth microdilution against common skin-relevant bacteria, with MICs of 250 µg/mL for *Staphylococcus aureus* and 500 µg/mL for *Escherichia coli*, alongside additional activity testing against *Bacillus subtilis* and *Pseudomonas aeruginosa*, indicating a broad antibacterial profile in the screening panel. Dermal safety testing aligned with OECD 404 suggested low acute irritation potential: topical application produced only slight, transient erythema at 1 h (median 0.5 ± 0.5) that resolved by 24 h, with no edema observed. Finally, in vivo anti-inflammatory efficacy was supported using a carrageenan-induced rat paw edema model (topical dosing post-induction), where the nanoemulgel reduced edema relative to blank gel at early time points, reported inhibition of 40.5% (30 min), 60.3% (60 min), 81.6% (120 min), and 96.9% (240 min), with complete edema resolution by 360 min in the treated group, consistent with a pharmacodynamically meaningful anti-inflammatory effect compatible with topical management of inflammatory skin conditions [131].

Although the preclinical literature supports multiple mechanistic hypotheses, clinical translation requires that these signals persist at human-relevant exposures and in designs that control expectancy, co-interventions, and outcome multiplicity.

## 5. Clinical Evidence and Translational Considerations

Accordingly, the clinical evidence is summarized below by indication and outcome type, emphasizing effect direction and magnitude where available and explicitly noting design features (e.g., multi-ingredient formulations or non-randomized comparators) that constrain attribution to lemon balm alone and limit generalizability.

### 5.1. Anxiety, Stress and Cognition Domain

In a study conducted by Bagheri Motahareh et al., eighty adults scheduled for orthopedic surgery were randomized to receive *Melissa officinalis* leaf extract. The extraction protocol was hydroalcoholic maceration, followed by separation and drying after maceration. The mixture was filtered; then the supernatant was dried in an oven at 40 °C to obtain the crude extract, and the extract was subsequently standardized for phenolics. Participants in the intervention arm received two doses of 500 mg extract capsules (at 4 pm and 10 pm on the day before surgery), while controls received identical placebo capsules (starch) on the same schedule. Baseline anxiety did not differ between groups, but preoperative anxiety on the surgery day was significantly lower in the Melissa group (mean anxiety 44.01 ± 6.98 to 35.94 ± 11.56) compared with placebo (45.17 ± 9.11 to 43.35 ± 7.33; inter-group *p* = 0.007) [132]. In another study conducted by Lotfi et al., ninety-six coronary care unit (CCU) patients were randomized to aromatherapy with *Melissa officinalis* extract versus a control condition using odorless sesame oil. The intervention used a 15 cm × 15 cm cotton patch containing three drops of lemon balm extract affixed to the patient’s collar for 30 min, twice daily for three days, and anxiety was measured using a Spielberger-based questionnaire at baseline, during treatment, and post-intervention. Baseline anxiety was comparable between groups (pretest mean ± SD: control 90.14 ± 18.76 versus intervention 89.62 ± 16.79, *p* > 0.05), and there was no statistically significant difference during treatment (control 88.70 ± 22.62 versus intervention 83.51 ± 15.89, *p* > 0.05). By the post-test, however, the intervention group showed a significantly lower total anxiety score than controls (control 88.46 ± 18.85 versus intervention 80.24 ± 13.63, *p* < 0.05), supporting an anxiolytic signal for short-course lemon balm aromatherapy in this acute cardiac-care context [133].

Sinaei et al. performed a randomized clinical trial in fifty mothers of preterm infants in the NICU to test whether inhalation aromatherapy with *Melissa officinalis* (lemon balm) essential oil improves sleep quality, using the Pittsburgh Sleep Quality Index (PSQI) measured at baseline, 1 week, and 2 weeks. The intervention consisted of applying 5 to 7 drops of lemon balm essential oil to a face mask twice daily for two weeks, while the control group received routine care. Baseline PSQI scores were comparable between groups, but the intervention group showed significantly greater improvements thereafter, with mean PSQI decreasing to 8.60 ± 2.28 at week 1 and 5.20 ± 1.87 at week 2, compared with 11.08 ± 2.73 and 10.68 ± 2.88 in controls at the same time points, respectively, indicating a marked, time-dependent improvement in subjective sleep quality with between-group differences reported as highly significant (*p* < 0.001) [134].

Bano et al. reported results from a 3-week, prospective, randomized, double-blind, placebo-controlled parallel trial in 100 healthy adults with moderate emotional distress and/or poor sleep, testing a standardized phospholipid carrier-based *Melissa officinalis* aqueous extract (Relissa^™^; leaf aqueous extract standardized to 17–23% hydroxycinnamic acid derivatives) 200 mg twice daily (400 mg/day) versus placebo. Outcomes included DASS-42 (depression/anxiety/stress), PSQI, WEMWBS, PANAS, and WHO-QoL-BREF. At follow-up, the Melissa group showed markedly larger improvements than placebo across all primary distress endpoints on DASS-42, with significant group time interactions (all *p* < 0.001): depression decreased from 23.3 ± 7.9 to 6.9 ± 4.5 versus placebo 22.7 ± 8.1 to 17.1 ± 8.7; anxiety decreased from 22.2 ± 7.2 to 6.7 ± 3.8 versus 21.1 ± 8.0 to 15.9 ± 7.3; and stress decreased from 26.3 ± 7.7 to 8.2 ± 4.8 versus 26.1 ± 6.9 to 20.0 ± 9.4. Mental wellbeing also improved more with Melissa (WEMWBS 34.5 ± 9.5 to 50.0 ± 9.0) than placebo (37.8 ± 8.3 to 41.1 ± 9.8) with group time *p* < 0.001, alongside improvements in affect (PANAS positive affect 12.9 ± 3.2 to 18.0 ± 3.0 versus 13.8 ± 3.0 to 14.8 ± 3.4; PANAS negative affect 18.2 ± 3.1 to 11.0 ± 3.2 versus 16.5 ± 4.6 to 15.0 ± 4.6; group time *p* < 0.001). Sleep quality (PSQI) improved substantially in the Melissa group (10.9 ± 4.3 to 3.5 ± 2.6) compared with placebo (8.6 ± 3.8 to 6.6 ± 3.3), with a significant group time interaction (*p* < 0.001), although baseline PSQI differed between groups (*p* = 0.006), which is important for interpretation. Quality of life also improved across WHO-QoL-BREF domains with significant group time effects (all *p* < 0.001), including total QoL (5.0 ± 1.3 to 7.3 ± 1.3 versus placebo 6.0 ± 1.4 to 6.4 ± 1.5), and the extract was well tolerated, with only mild stomach upset reported in both arms and no serious adverse events [135].

Pasyar et al. conducted a randomized clinical trial in 68 hemodialysis patients to test whether inhalation aromatherapy with *Melissa officinalis* essential oil reduces anxiety and symptom burden during dialysis, compared with placebo aromatherapy using refined sweet almond oil. The intervention used 200 µL essential oil applied to a cotton ball pinned to clothing approximately 20 cm from the nose and inhaled for 20 min during the first 20 min of hemodialysis, three times per week for one month. Total DSI severity decreased within the Melissa group (53.08 ± 22.18 to 41.50 ± 21.32; within-group *p* < 0.001), but the post-intervention between-group difference in total DSI score was not statistically significant (*p* = 0.16), suggesting that the most robust and clearly separable treatment effect was on anxiety outcomes rather than overall symptom burden in this sample. At the symptom level, post-intervention between-group differences favored Melissa for the severity of constipation, nausea, vomiting, diarrhea, muscle cramps, swelling in legs, muscle “shortness,” worrying, nervousness/anxiety, and trouble staying asleep, and for symptom prevalence specifically for feeling anxious and dry mouth (*p* < 0.05), supporting a targeted symptom-improvement profile despite the non-significant between-group difference in overall Dialysis Symptom Index total score [136].

Noguchi-Shinohara et al. conducted a randomized, double-blind, placebo-controlled 24-week trial in 23 patients with mild dementia due to Alzheimer’s disease, testing *Melissa officinalis* extract providing 500 mg/day rosmarinic acid (primary outcome: safety/tolerability). While cognitive and functional measures (e.g., ADAS-cog, MMSE, DAD, CDR) and several biomarkers did not show statistically significant differences between groups during the blinded phase, the authors reported a significant improvement in neuropsychiatric symptoms on the NPI-Q with treatment, with mean NPI-Q change indicating improvement in the Melissa group (−0.5 ± 1.9) versus worsening in placebo (+0.7 ± 1.0). A domain-level signal was also reported for irritability/lability. Adverse events occurred at similar frequencies across groups and were not serious or trial-limiting [137].

Shirazi et al. conducted an 8-week, randomized, double-blind, placebo-controlled, three-arm trial in 60 postmenopausal women with sleep disturbance, comparing a capsule described as a combination of lemon balm leaf with fennel fruit extract 500 mg/day, citalopram 30 mg/day, and placebo, with menopausal quality of life assessed by MENQOL, where higher scores indicate worse quality of life. Relative to both comparators, the authors reported statistically significant between-group differences across all MENQOL domains (*p* < 0.001) and the largest improvements in the Melissa-containing arm when change from baseline was considered, with post-intervention mean ± SD domain scores reported as follows for the Melissa-containing, citalopram, and placebo groups, respectively: vasomotor 2.2 ± 0.84, 0.56 ± 0.58, and 0.36 ± 0.55; psychosocial (psychomotor-social) 1.02 ± 0.60, 0.28 ± 0.20, and 0.17 ± 0.10; physical 0.76 ± 0.40, 0.25 ± 0.10, and 0.11 ± 0.10; and sexual 2.3 ± 1.0, 0.35 ± 0.5, and 0.41 ± 0.5, alongside reported percentage improvements that were greatest in the Melissa-containing arm across domains. Adverse events were reported in the citalopram group (nausea, headache), while none were reported in the Melissa-containing or placebo groups. Because the tested intervention combined lemon balm with fennel, the observed effects cannot be attributed to *Melissa officinalis* alone [138].

Alvarado-García et al. conducted a quasi-experimental, waiting-list controlled study in which participants self-administered *Melissa officinalis* essential oil inhalation daily for 6 weeks, using a cotton ball with essential oil held under the nose while taking 10 deep breaths and then pinned to the collar for 30 min, while the waiting-list group received no intervention. State anxiety decreased significantly within the experimental group, with State-Trait Anxiety Inventory (STAI)-state scores declining from 35.76 ± 6.21 at baseline to 30.50 ± 6.81 post-intervention (within-group *p* = 0.007), whereas the waiting-list group showed no meaningful change, and post-test between-group differences favored the experimental condition (*p* = 0.008). In contrast, trait anxiety did not change significantly in the experimental group (*p* = 0.192) and did not differ between groups at post-test (*p* = 0.895), suggesting that the intervention’s detectable signal was confined to situational anxiety rather than stable anxiety disposition. Interpretation is constrained by the non-randomized design, limited blinding, and the use of a waiting-list comparator, which together limit causal inference and increase susceptibility to expectancy and contextual effects [139].

### 5.2. Dermatology

Yargholi et al. conducted a 12-week, randomized, double-blind, placebo-controlled clinical trial in 100 adults with mild-to-moderate plaque psoriasis, testing an oral traditional persian medicine syrup containing lemon balm (3 g), Damask rose (8 g), fennel (1 g), and honey versus a matched placebo (dose: two tablespoons 10 mL each; three times daily before meals). Compared with placebo, the syrup group showed significantly greater improvement in both itch intensity and disease severity: mean pruritus (VAS) decreased from 6.34 ± 2.68 at baseline to 3.45 ± 2.18 at week 12 (within-group *p* < 0.001), whereas placebo showed no significant change (baseline 5.45 ± 2.55; week 12: 5.56 ± 2.81; *p* = 0.886), and the between-group difference at endpoint was significant (*p* < 0.001). PASI similarly improved in the intervention group from 3.26 ± 3.58 to 1.65 ± 2.17 (*p* < 0.001), while placebo worsened from 2.95 ± 2.16 to 3.36 ± 2.38 (*p* = 0.002), yielding a significant between-group endpoint difference (*p* < 0.001). Quality-of-life scores increased within the intervention group (*p* = 0.029), but the between-group difference at study end was not statistically significant (*p* = 0.065), suggesting that symptomatic and PASI improvements were not mirrored by a statistically robust advantage under the study conditions. Also, because the syrup is a multi-ingredient formulation, these clinical effects cannot be attributed to *Melissa officinalis* alone [140].

### 5.3. Metabolic Outcomes

Dolatkhah et al. conducted a double-blind randomized controlled trial in adults with type 2 diabetes and symptoms consistent with diabetic neuropathy (64 randomized; 60 completed, 30 per arm) to evaluate *Melissa officinalis* capsules over 3 months, using fasting blood glucose (FBS) as the primary endpoint and nerve-conduction parameters as secondary outcomes. Post-intervention, the Melissa group showed a significantly greater reduction in FBS compared with placebo (*p* = 0.02), whereas HbA1c did not differ significantly between groups at study end (*p* = 0.08). On electrodiagnostic outcomes, the intervention was associated with improvements in selected lower-limb nerve parameters, including a significant increase in tibial motor nerve action potential amplitude (*p* = 0.001) and higher tibial motor nerve conduction velocity at post-test (*p* = 0.03), along with significant intra-group or change-score differences favoring the intervention for peroneal motor nerve action potential amplitude (*p* = 0.002) and sural sensory nerve potential amplitude (between-group difference after intervention *p* = 0.009, with change-score difference *p* = 0.01). In contrast, there were no significant effects on ulnar and median nerve parameters or on F-wave latency, suggesting that measurable benefits were limited to specific peripheral nerve readouts under the study conditions [141].

In a double-blind, randomized, placebo-controlled trial in adults with type 2 diabetes and depressive symptoms (n = 60 randomized; n = 44 completers), Safari et al. administered *Melissa officinalis* hydroalcoholic extract at 700 mg/day for 12 weeks and found clinically relevant improvements in mood-related endpoints compared with placebo. In completers, the Melissa group showed significantly greater improvements in mood outcomes than placebo, with beck depression inventory (BDI)-II decreasing from 18.00 ± 10.30 at baseline to 14.08 ± 7.63 at week 12, while placebo remained essentially unchanged (14.28 ± 5.11 to 14.23 ± 5.43), yielding a significant between-group difference in mean change (*p* = 0.001). Beck Anxiety Inventory (BAI) similarly improved in the Melissa arm (11.82 ± 8.10 to 9.78 ± 7.60) compared with placebo (8.09 ± 5.32 to 8.33 ± 5.69), with a significant between-group difference in mean change (*p* = 0.04). In contrast, there were no significant between-group differences for fasting blood sugar, hs-CRP, anthropometrics, blood pressure, or sleep quality (PSQI mean-change comparison *p* = 0.15), and no adverse effects were reported. The extract was chemically characterized, with total flavonoids reported as 148.06 mg rutin equivalents per gram and rosmarinic acid quantified at 8.10 ± 0.04 mg per capsule, supporting interpretability of the intervention content [142].

### 5.4. Oncology Domain

Another study conducted by Ehsani et al., cancer patients with chemotherapy-induced peripheral neuropathy (CIPN) were randomized in a double-blind design to receive gabapentin (300 mg/day) plus *Melissa officinalis* hydroalcoholic extract (500 mg twice daily) or gabapentin plus placebo for 3 months. Neuropathy severity was assessed using CTCAE, and quality of life using EORTC QLQ-C30. At study end, CTCAE neuropathy severity differed significantly between groups (final CTCAE distribution, *p* = 0.010), indicating that adding lemon balm altered neuropathy outcomes relative to placebo under otherwise similar management. Overall, the trial supports a modest adjunctive signal for lemon balm when combined with Gabapentin, particularly for pain perception and gastrointestinal symptom burden, with gastrointestinal intolerance being the main reported limitation affecting adherence in a minority of participants [143].

### 5.5. Other Clinical Outcomes

Naderi Dastjerdi et al. conducted a randomized, single-blind clinical trial in 110 women with moderate-to-severe postpartum after-pains, comparing *Melissa officinalis* extract capsules (395 mg pure extract every 6 h for 24 h; four doses) with mefenamic acid (250 mg every 6 h for 24 h). Pain severity (0–10 numeric scale) was measured repeatedly before and after each dose. Baseline pain severity was comparable between groups (6.44 ± 0.91 versus 6.22 ± 1.01; *p* = 0.23). After the first dose, pain did not differ at 1 h or 2 h but became significantly lower in the Melissa group at 3 h (3.38 ± 1.06 versus 3.94 ± 1.24; *p* = 0.01). More pronounced between-group separation emerged after subsequent doses, with significantly lower pain in the Melissa group one hour after the second dose (3.55 ± 0.83 versus 4.30 ± 0.98; *p* < 0.001), one hour after the third dose (3.02 ± 0.99 versus 3.95 ± 0.95; *p* < 0.001), and one hour after the fourth dose (2.54 ± 0.80 versus 3.65 ± 0.96; *p* < 0.001) [144].

Alijaniha et al. describe a pilot randomized, double-blind, placebo-controlled clinical trial protocol (n = 20) evaluating *Melissa officinalis* (500 mg twice daily) for 12 weeks in moderate rheumatoid arthritis, with primary outcomes including disease activity indices (e.g., DAS28-ESR/CRP) and inflammatory biomarkers (TNF-α, IL-17). As this is a protocol paper, it outlines planned methods and outcomes but does not yet provide clinical efficacy results [145].

Zare Javid et al. conducted a 2-month, double-blind, randomized trial in 80 patients with chronic stable angina comparing powdered *Melissa officinalis* at 3 g/day (1 g three times daily) with placebo and reported multi-domain improvements in cardiac functional indices and related biomarkers. Post-intervention, the Melissa group showed an increase in echocardiographic ejection fraction from 46.0 ± 4.97 to 48.86 ± 3.85, while the placebo group changed minimally from 48.29 ± 4.54 to 48.60 ± 4.81, and exercise capacity improved with maximum workload rising from 8.60 ± 1.57 to 10.41 ± 1.89 metabolic equivalents (METs) versus no improvement in placebo (9.99 ± 2.27 to 9.92 ± 2.43), with between-group differences reported as significant (*p* < 0.01 to *p* < 0.001 depending on endpoint). Biochemically, serum lactate dehydrogenase (LDH) decreased in the Melissa arm from 308.23 ± 72.06 to 251.86 ± 77.37 U/L while increasing in placebo from 282.45 ± 56.32 to 289.34 ± 63.09 U/L, and nitric oxide increased in the Melissa group from 20.51 ± 13.88 to 28.64 ± 15.77 μmol/L while decreasing in placebo from 20.61 ± 8.41 to 19.77 ± 8.09, again with significant between-group differences (reported *p* < 0.001). Hemodynamic outcomes also favored the intervention, with systolic blood pressure (SBP) decreasing to 119.41 ± 4.54 mmHg versus 122.32 ± 5.28 mmHg in placebo (between-group *p* < 0.001) and diastolic blood pressure (DBP) decreasing to 77.57 ± 5.86 mmHg versus 79.86 ± 4.10 mmHg (between-group *p* = 0.017), supporting a short-term cardiometabolic benefit signal in this population [146].

### 5.6. Limitations and Translational Outcomes

Across the available clinical literature, *Melissa officinalis* interventions show the most consistent signals in symptom-centered outcomes, particularly anxiety, stress, and sleep quality, with effects observed across different delivery formats. These studies are supportive of a clinical benefit profile that is plausible and dependent on preparation, but interpretation is hindered by heterogeneous formulations/standardization and frequent multi-ingredient interventions.

The review synthesis indicates that translation of *Melissa officinalis* research is primarily limited by the mismatch between botanical labeling and chemical exposure. Lemon balm interventions vary substantially between polyphenol-rich extracts, often standardized to rosmarinic acid, and essential oil preparations dominated by volatile terpenoids, which likely engage partially distinct biological pathways. This variability complicates cross-study comparison and undermines mechanistic inference unless minimum reporting standards are adopted. A second translational barrier is dose and exposure plausibility, because many in vitro concentrations and short-duration animal paradigms may not reflect achievable human pharmacokinetics. In clinical research, translational value is further reduced by fragmented trial designs, inconsistent outcome instruments, and frequent multi-ingredient formulations that limit attribution to *M. officinalis*; therefore, future trials should prioritize chemically characterized single-ingredient preparations, harmonized clinically interpretable outcomes, and standardized safety reporting.

## 6. Research Gaps and Future Directions

Despite the breadth of reported bioactivities, the strongest limitation across the lemon balm literature is the persistent mismatch between “plant name” and “chemical identity”, because many studies describe lemon balm interventions without sufficient characterization to enable reproducibility or cross-study comparison. Future work should adopt minimum reporting standards that include plant part, extraction method, marker compounds (e.g., rosmarinic acid content for extracts; citral isomer ratio and major terpenoids for essential oils), and batch-to-batch variability. Also, much of the available evidence was generated in Mediterranean/European contexts, which may limit how directly findings extrapolate to other populations and patterns of use. Regional differences in cultivation conditions, processing traditions, chemotype, and background lifestyle factors may influence both phytochemical composition and observed outcomes. Broader geographic sampling and clearer reporting of plant provenance/chemotype and preparation standardization would strengthen external validity and support more robust evidence synthesis.

A second gap concerns pharmacokinetic and dose-translation plausibility. Many preclinical effects are demonstrated in vitro at concentrations that may not be achievable systemically in humans, while animal studies often employ short durations and exposure routes that do not map cleanly onto dietary or supplement use. Bridging studies are needed to quantify exposure for key marker compounds and to relate these exposures to mechanistic endpoints that are measurable across systems, such as oxidative-stress signatures, inflammatory mediators, endothelial/nitric oxide (NO)-related markers, or neurotrophic correlates highlighted in the current synthesis. Where feasible, physiologically based pharmacokinetic reasoning and standardized dose reporting (mg/kg of extract, mg/kg of marker compound) would reduce uncertainty around “active range” and better justify clinical dosing.

In clinical research, the principal weakness is fragmentation by: (i) small trials; (ii) variable blinding intensity; (iii) inconsistent outcome instruments; and (iv) frequent multi-ingredient formulations complicate attribution to *M. officinalis* alone. Several studies summarized in the manuscript report improvements in symptom-focused outcomes, but the comparators range from placebo oils to waiting-list controls, and some interventions combine lemon balm with other botanicals or carriers, limiting causal inference and external validity. Future trials should preferentially use chemically standardized single-ingredient preparations (or factorial designs if combinations are clinically intended), pre-register primary outcomes, and apply consistent instruments and clinically interpretable effect-size reporting to support evidence synthesis and guideline relevance. Across the clinical studies reviewed, *Melissa officinalis* interventions were generally well tolerated, and reported adverse events were typically mild and transient when described; however, safety reporting was inconsistent across trials and follow-up durations were often limited. Given the variability in formulations (single-ingredient versus multi-ingredient; extracts versus essential oils), standardized adverse-event reporting and clearer documentation of concomitant medications are needed. Also, future clinical programs should incorporate standardized safety endpoints, longer follow-up where chronic use is plausible, and stratification by relevant comorbidities and co-medications to better define risk-benefit boundaries.

## 7. Conclusions

Overall, the evidence synthesized in this review supports *Melissa officinalis* as a chemically diverse Lamiaceae species whose bioactivity is plausibly driven by a preparation-dependent balance between polyphenol-rich fractions and volatile terpenoid constituents typical of essential oils. Across preclinical domains, lemon balm preparations are repeatedly associated with antioxidant and anti-inflammatory signatures and with downstream functional effects that vary by model system, dosing, and standardization, reinforcing the central interpretive theme that lemon balm is not a single exposure but a family of chemically distinct interventions. Clinically, the emerging literature summarized in the manuscript suggests that lemon balm-containing interventions may improve selected symptom-oriented endpoints in certain contexts (e.g., anxiety/stress and sleep-related outcomes), but conclusions are limited by heterogeneous (often multi-ingredient) formulations and variable study designs, which restrict attribution and comparability. Consequently, while the collective findings justify continued investigation and formulation development, translation to practice-level recommendations requires a tighter methodological standard centered on chemical standardization, dose and exposure plausibility, harmonized outcomes, and adequately powered, well-controlled trials with transparent safety reporting.

## Figures and Tables

**Table 1:** Some of the representative medicinal and aromatic species of the Lamiaceae family.

Genus	Species	Phytochemistry	Biological Activities	References
*Ajuga*	*Ajuga* *reptans*	Phenolic compounds (e.g., hydroxycinnamic acids, flavonoids, tannins), iridoids, terpenoids, saponins, phytosterols, organic acids, amino acids	Hepatoprotective activity Anti-inflammatory activity Hemostatic and wound-healing activity Antimicrobial activity	[11,12]
	*Ajuga* *genevensis*	Iridoids, polyphenolic compounds, phytosterols	Antioxidant activity Antimicrobial activity Antifungal activity Anti-inflammatory activity (in vivo) Anticancer effects (in vitro)	[13,14]
*Teucrium*	*Teucrium* *pruinosum*	Iridoids, terpenoids, flavonoids	Anti-inflammatory (COX inhibition, essential oil) Antioxidant activity Cytotoxic activity (in vitro on HeLa; essential oil)	[15,16]
*Scutellaria*	*Scutellaria* *galericulata*	Flavonoids, terpenoids	Antimicrobial activity Anti-inflammatory activity Antiviral activity	[17,18,19]
	*Scutellaria* *hastifolia*			
	*Scutellaria* *grossa*			
	*Scutellaria* *barbosa*			
	*Scutellaria* *lateriflora*			
	*Scutellaria* *altissima*			
	*Scutellaria* *alpina*			
*Lavandula*	*Lavandula* *angustifolia*	Terpenes, phenolic acids and flavonoids	Anxiolytic/antidepressant-relevant CNS activity Antioxidant activity Anti-inflammatory activity Antimicrobial activity Neuroprotective potential	[20,21,22,23,24,25]
*Prunella*	*Prunella* *vulgaris*	Triterpenoids, steroids, flavonoids, organic acids, volatile oils, polysaccharides, tannins	Anti-inflammatory activity Antioxidant activity Antibacterial and antiviral activities Anti-aging and immunomodulatory profile Anti-herpes simplex virus activity Anti-hyperlipidemic activity	[26,27,28,29]
*Lamium*	*Lamium* *maculatum*	Essential oils, hydroxycinnamic acids, iridoids–secoiridoids, flavonoids, anthocyanins, phenylpropanoids, phytoecdysteroids	Antispasmodic activity Anti-inflammatory activity Anti-proliferative effect Antioxidant activity Wound-healing properties	[30,31,32]
	*Lamium* *album*			
	*Lamium* *purpureum*			
*Stachys*	*Stachys* *sylvatica*	Terpenes (e.g., triterpenes, diterpenes, iridoids), polyphenols (e.g., flavone derivatives, phenylethanoid glycosides, lignans), phenolic acids, tannins, essential oils	Antioxidant activity Antimicrobial activity Antiproliferative (anticancer-relevant) activity Anthelmintic and antiviral signals	[33,34,35,36,37,38,39]
	*Stachys* *recta*			
	*Stachys* *arvensis*			
	*Stachys* *palustris*			
*Thymus*	*Thymus* sp.	Terpenoids, phenolic acids, flavonoids	Antimicrobial activity Antioxidant activity Anti-inflammatory activity	[40,41,42,43,44,45,46]
*Origanum*	*Origanum* *vulgare*	Acyclic, monocyclic (e.g., thymol, carvacrol) and bicyclic monoterpenes, flavonoids (e.g., luteolin, apigenin, quercetin), phenolic acids (e.g., rosmarinic and chlorogenic acids), tannins	Antimicrobial activity Antioxidant activity Anti-inflammatory activity Anticancer activity Antiangiogenic effect	[47,48,49,50,51]
*Mentha*	*Mentha x piperita*	Phenolic acids, flavonoids, monoterpenoids	Antimicrobial activity Anti-inflammatory activity Antiviral activity Immunomodulatory activity Anticancer activity Neuroprotective effect Cognitive-enhancing effects Hypoglycemic and hypolipidemic effects Wound-healing effect Antispasmodic activity Antihelmintic activity	[52,53,54,55]
*Ocimum*	*Ocimum* *basilicum*	Terpenoids, phenolic acids, flavonoids, tannins, saponins, glycosides (e.g., cardiac glycosides), steroids	Antimicrobial activity Antioxidant activity Anti-inflammatory activity Antiviral activity Wound-healing effect Immunomodulatory effect Anticancer activity Antihypertensive activity	[56,57,58,59,60,61]
	*Ocimum* *tenuiflorum*	Terpenoids, flavonoids, phenolic acids	Antioxidant, neuroprotective, anticancer, antidiabetic, antipyretic, and anti-inflammatory activities Hepatoprotective and cardioprotective effects	[62]
*Salvia*	*Salvia* *hispanica*	ω-3 fatty acids, phenolic acids, flavonoids (e.g., myricetin, quercetin, kaempferol), phytosterols, amino acids (e.g., L-phenylalanine, L-tryptophan)	Antioxidant, antimicrobial, anti-inflammatory, and antidiabetic activities Cholinesterase inhibition	[63,64]
	*Salvia* *nemorosa*	Phenolic acids (e.g., rosmarinic acid, sagerinic acid, caffeoylquinic acids), flavonoids, fatty acyl glycosides, monoterpenes (e.g., α-pinene, β-pinene, limonene, myrcene)	Antimicrobial and antioxidant activities Enzyme inhibition (e.g., α-glucosidase, xanthine oxidase, aldehyde oxidase, acetylcholinesterase)	[65,66]
	*Salvia* *officinalis*	Terpenoids (e.g., borneol, camphor, 1,8-cineole, pinene, thujone, caryophyllene), phenolic acids (e.g., rosmarinic acid, caffeic acid, chlorogenic acid, ellagic acid)	Anticancer, anti-inflammatory, antinociceptive, antioxidant, antimicrobial, antimutagenic, antidementia, and antidiabetic activities Not recommended in pregnancy and lactation	[67,68]
	*Salvia rosmarinus* (syn. *Rosmarinus* *officinalis*)	Monoterpenes, phenolic acids (e.g., rosmarinic acid), abietane diterpenes, flavonoids	Anti-inflammatory, antimicrobial, antioxidant, anticholinesterase, antidiabetic, and anticancer activities Wound-healing effect	[69,70,71,72,73,74,75]
*Plectranthus*	*Plectranthus* *amboinicus*	Monoterpenes (e.g., carvacrol, thymol, p-cymene, γ-terpinene), flavones (e.g., cirsimaritin, salvigenin, chrysoeriol)	Antimicrobial, antioxidant, anti-inflammatory and anticancer potential activities Wound-healing effect	[76,77,78]
	*Plectranthus* *hadiensis*	Abietane-type diterpenes (e.g., royleanones)	Antioxidant and antimicrobial activities	[78,79]
	*Plectranthus* *ornatus*	Diterpenes, flavonoids	Anti-inflammatory and anticancer activities	[79,80,81,82,83]
*Leonurus*	*Leonurus cardiaca*	Flavonoids (e.g., quercetin, rutin, genkwanin, apigenin), iridoids, labdane diterpenes, p-hydroxycinnamic acid derivatives (e.g., ferulic, chlorogenic and caffeic acids)	Nutraceutical product Cardiovascular effect Anti-inflammatory activity Antimicrobial activity Antioxidant activity	[84,85]
*Satureja*	*Satureja* sp.	Essential oils (e.g., carvacrol, γ-terpinene, thymol, p-cymene), fatty acids (e.g., linolenic (ω-3), palmitic, linoleic (ω-6), oleic (ω-9), stearic, and palmitoleic (ω-7) acids)	Anticholinergic, antioxidant, cytotoxic, antimicrobial, anti-inflammatory, analgesic, and antifungal activities	[86]

## Data Availability

The original contributions presented in the study are included; further inquiries can be directed to the corresponding author.
